# Deletion of the L7L-L11L Genes Attenuates ASFV and Induces Protection against Homologous Challenge

**DOI:** 10.3390/v13020255

**Published:** 2021-02-08

**Authors:** Jingyuan Zhang, Yanyan Zhang, Teng Chen, Jinjin Yang, Huixian Yue, Lidong Wang, Xintao Zhou, Yu Qi, Xun Han, Junnan Ke, Shuchao Wang, Jinmei Yang, Faming Miao, Shoufeng Zhang, Fei Zhang, Ying Wang, Min Li, Rongliang Hu

**Affiliations:** 1 College of Life Sciences, Ningxia University, Yinchuan 130006, China; zjyuanff27@163.com (J.Z.); zhouxtao@foxmail.com (X.Z.); 2 Veterinary Research Institute, Institute of Military Medical Sciences, Academy of Military Sciences, Changchun 130122, China; Yanyanzhang90615@163.com (Y.Z.); ctcx1991@163.com (T.C.); jjyang2021@163.com (J.Y.); 18380384852@163.com (H.Y.); 15164390097@163.com (L.W.); qiyu0204@163.com (Y.Q.); hx19871111@126.com (X.H.); kejunnan0125@163.com (J.K.); wsc1026@126.com (S.W.); charminggirlyjm@163.com (J.Y.); miaofaming81@163.com (F.M.); zhangshoufeng@hotmail.com (S.Z.); fei2333@163.com (F.Z.); wy97025@126.com (Y.W.)

**Keywords:** African swine fever virus, L7L-L11L genes, deletion, vaccine candidate

## Abstract

African swine fever (ASF), caused by the African swine fever virus (ASFV), is a major epidemic disease endangering the swine industry. Although a number of vaccine candidates have been reported, none are commercially available yet. To explore the effect of unknown genes on the biological characteristics of ASFV and the possibility of a gene-deleted isolate as a vaccine candidate, the strain SY18ΔL7-11, with deletions of L7L–L11L genes from ASFV SY18, was constructed, and its biological properties were analyzed. The results show that deletion of genes L7L-L11L did not affect replication of the virus in vitro. Virulence of SY18△L7-11 was significantly reduced, as 11 of the 12 pigs survived for 28 days after intramuscular inoculation with a low dose (10^3^ TCID_50_) or a high dose (10^6^ TCID_50_) of SY18ΔL7-11. All 11 surviving pigs were completely protected against challenge with the parental ASFV SY18 on 28 days postinoculation (dpi). Transient fever and/or irregularly low levels of genomic DNA in the blood were monitored in some pigs after inoculation. No ASF clinical signs or viremia were monitored after challenge. Antibodies to ASFV were induced in all pigs from 14 to 21 days postinoculation. IFN-γ was detected in most of the inoculated pigs, which is usually inhibited in ASFV-infected pigs. Overall, the results demonstrate that SY18ΔL7-11 is a candidate for further constructing safer vaccine(s), with better joint deletions of other gene(s) related to virulence.

## 1. Introduction

African swine fever (ASF) is a highly contagious hemorrhagic disease, causing a wide spectrum of clinical syndromes ranging from mild to high lethality, with a virulent strain inducing great loss to the swine industry. Its pathogen, the African swine fever virus (ASFV), is a DNA virus and the only member of the genus *Asfivirus*, family *Asfarviridae*. Based on variations of the p72 gene sequence (open reading frame (ORF) B646L) of ASFV, 24 genotypes have been identified, of which genotypes I, II, and IX are among the most widely distributed [1,2,3,4], and genotype II has spread to wider regions including Europe, the Far East, West Asia, and neighboring countries since its introduction from East Africa into the Caucasus region of Georgia in 2007. In August 2018, ASF of genotype II was first confirmed in China [5]. Later, it spread to other pig-raising countries in Asia and Oceania [6,7].

Quarantine and slaughter are the main strategies to prevent, control, and eliminate ASF. No ASF vaccine has been approved so far due to the complexity of the virus and the mechanism of its immunity. In recent years, it has been demonstrated that several naturally attenuated strains and genetically engineered strains with different virulence-related gene deletions show an immunoprotective effect on swine against virulent virus challenge [8,9,10,11,12,13]. However, these studies have not been approved for release yet. It has been found that some naturally attenuated and engineered ASFV induce side effects, such as skin ulcer, persistent fever, viremia, hyperimmunoglobulinemia and/or joint swelling, conjunctivitis, abortion, and so forth, in field trials [14].

ASFV has a 170–190 kb genome containing 160–175 open reading frames (ORFs) and encoding more than 160 proteins, only a small part of which has been studied and functionally characterized. The ORFs of L7L–L11L (containing L7L, L8L, L9R, L10L, and L11L, abbreviated as L7L-L11L) are clustered and located at the right variable region of the ASFV genome [15]. Descriptions about the functions of these genes are quite limited. L7L and L8L are considered to be members of MGF100 [16], while the role of their coding products has not been clarified. L10L was reported to be homologous with the KP177R gene (encoding the p22 protein) at the left end of the genome. Deletion of L11L did not affect the virulence of strain Malawi Lil-20 in vivo [17]. L9R is an unknown gene located between L8L and L10L. More importantly, none of the five proteins have been found in viral particles. BA71V is an avirulent ASFV strain from BA71 obtained during adaption to Vero cells. However, the avirulent strain was found to possess four large fragment deletions, and one of the fragments affects the above five genes plus DP148R. Whether the loss of this fragment is related to its virulence attenuation is unclear [18]. A Georgia strain (ASFV-G) was also found to have deletion of this fragment together with other regions during adaptation to Vero cells, and this deletion was found to be partially reversible [19]. We wonder if deletion of the L7L-L11L fragment merely would affect the virulence of ASFV, the immunity, and the host response to the virus so as to develop a vaccine candidate. Therefore, we deleted the L7L-L11L genomic fragment of ASFV isolate SY18 and tested its replication ability in vitro, its virulence in swine, and its virological properties.

## 2. Materials and Methods

### 2.1. Cells and Viruses

Primary bone marrow-derived macrophages (BMDMs) were prepared from 2 to 3 month old piglets. Briefly, bone marrow was collected from medullary cavities. After lysis of erythrocytes with—red blood cell lysis buffer (TBD, Tianjin, China) and rinsing with phosphate-buffered saline (PBS), the cells were resuspended and grown in RPMI 1640 (Gibco, Beijing, China) supplemented with 10% fetal bovine serum (Gibco) and 10 ng/mL GM-CSF. Cells were cultured in an incubator at 37 °C under 5% CO_2_. Part of the cell sample was taken and submitted for ASFV, classical swine fever virus (CSFV), porcine reproductive and respiratory syndrome (PRRSV), porcine pseudorabies virus (PRV), porcine parvovirus (PPV), and porcine circovirus (PCV)1/2 assay using methods of national standards. The primers are displayed in Table S1 (see Supplementary Materials).

The ASFV SY18 strain, GenBank no. MH766894.2, was isolated from swine specimens of the initial outbreak in China in 2018 by Epidemiology Laboratory of the Military Veterinary Research Institute. The fourth-generation virus was used in this study and stored at −80 °C in a biosecurity level 3 lab.

Virus titration was performed on BMDMs in 96-well plates (Corning, Wujiang, China) via an immunofluorescence assay using monoclonal antibody to ASFV protein p30. Briefly, virus cultures were diluted by a 10-fold gradient and inoculated on the monolayer cells. Five days after inoculation, cells were fixed using 80% acetone and incubated with fluorescein isothiocyanate (FITC)-labeled monoclonal antibody to p30 for 1 h at 37 °C, followed by observation under a fluorescence microscope. The titer of the virus was calculated by the Reed–Muench method.

### 2.2. Construction of Recombinant ASFV SY18△L7-11

Recombinant ASFV was generated by homologous recombination referring to the method previously reported with minor modifications [12]. The homologous recombinant transfer vector p△L7L-L11L-EGFP was constructed, which contained about 1.2 kb of the sequence to the left of L7L gene in the genome (Larm) and about 1.2 kb of the sequence to the right of the L11L gene (Rarm), and an EGFP gene was used as a fluorescence reporter under the control of the p72 promoter. BMDMs in 12-well plates (Corning) were transfected with 2 μg of p△L7L-L11L-EGFP using jetPEI®-macrophage transfection (Polyplus, Illkirch, France) and infected with ASFV SY18 at a multiplicity of infection (MOI) of 1.0. Fluorescent cells were selected and rinsed, followed by being distributed into healthy BMDMs in 96-well plates. After 5–6 rounds of monoclone selection, the recombinant virus was purified by an additional 3–5 rounds of limited dilution based on the fluorescent activity.

### 2.3. Polymerase Chain Reaction

A polymerase chain reaction (PCR) targeting the L7L-L11L fragment was performed to identify the purification of recombinant virus, using the following pair of primers: L7-F: 5′-TGGTAGTATTGTCCAAACCG-3′, L8-R: 5′-TAGGGACTTATGTAGTTTCGTC-3′. These primers were also used to assess the presence of the parental ASFV DNA in the blood of infected or challenged animals. EGFP was also detected using the following pair of primers: EGFP-F: 5′-CACCACCTGAATCTAATGAAG-3′, L11-R: 5′-ACACTAATGTGATGTCAAAT-3′.

### 2.4. Next-Generation Sequencing

To verify the accuracy of recombination, the full-length sequence of virus genomes was determined by next-generation sequencing. Total DNA was extracted from cell-cultured viruses and 1 μg of DNA was used for sequencing using Illumia novaseq6000, PE150 (Novogene Co., Ltd., Tianjin, China).

### 2.5. Viral Growth Curves

The preformed monolayer BMDMs in 24-well plates (Corning) were infected with the parental strain ASFV SY18 and the mutant virus ASFV SY18△L7-11, respectively, at an MOI of 0.1. After 1 h of adsorption at 37 °C under 5% CO_2_ and rinsing three times, the inoculum was discarded and replaced with RPMI 1640 culture medium containing 10% FBS. Incubation continued at 37 °C under 5% CO_2_. At appropriate time points of 2, 12, 24, 48, 72, and 96 h postinfection (hpi), cells were collected and titrated by the median tissue culture infectious dose (TCID_50_)/mL after freezing and thawing twice.

### 2.6. Animal Tests

Animal experiments were performed under animal biosecurity level 3 (ABSL-3) conditions, and they were approved by both the Animal Welfare and Ethics Committee of the Institute of Military Veterinary Medicine and the ABSL-3 lab (review ID: IACUC of AMMS-11-2019-018, approved at 1 November 2019). 

Pigs weighing 15–20 kg were obtained from a local farm with high biosecurity standards and hygiene. Common porcine viruses were tested via PCR or real-time PCR to ensure that animals were free of ASFV, CSFV, PRRSV, PRV, PPV, and PCV1/2.

Experiment 1: Virulence of SY18△L7-11.

Fifteen pigs were divided into three groups randomly. Six pigs in Group 1 (low-dose group), named SL1–SL6, were inoculated intramuscularly (i.m.) with 10^3^ TCID_50_ SY18△L7-11. Six pigs in Group 2 (high-dose group), named SH1–SH6, were inoculated with 10^6^ TCID_50_ SY18△L7-11 via the same route. Three pigs in Group 3 were inoculated intramuscularly (i.m.) with 10^3^ TCID_50_ ASFV SY18 as the virulent controls, named SW1–SW3. After inoculation, clinical manifestations were monitored, and rectal temperatures were recorded daily. Blood samples were collected at 0, 3, 7, 10, 14, 21, and 28 days postinoculation (dpi) for detection of ASFV nucleic acid, antibodies, and cytokines. 

Experiment 2: Protective effect of SY18△L7-11 against challenge of parental ASFV SY18.

At 28 days postinoculation, the surviving animals in Groups 1 and 2 (Experiment 1) were challenged with 10^3^ TCID_50_ ASFV SY18 intramuscularly (i.m.), and three healthy pigs that were not inoculated before, named SW4–SW6, were simultaneously challenged as none-immunized controls in Group 4. Clinical manifestations were monitored and rectal temperatures were recorded daily. Blood samples were collected at 0, 3, 7, 10, 14, and 21 days postchallenge (dpc) for detection of ASFV nucleic acid, antibodies, or cytokines. At the end of experiment, animals were euthanized using pentobarbital.

### 2.7. Quantitative PCR

Real-time quantitative PCR targeting the p72 gene of ASFV was used to quantify the ASFV genomic DNA copies in the blood of infected or challenged animals. Standard p72 plasmids were constructed by our laboratory. Forward primer 5′-CTGCTCATGGTATCAATCTTATCGA-3′, reverse primer 5′-GATACCACAAGATCAGCCGT-3′, and a taqman probe FAM-5′-CCACGGGAGGAATACCAACCCAGTG-3′-TAMRA were used. Amplification conditions used were a preheating at 95 °C for 30 s and 40 cycles of 95 °C for 5 s and 60 °C for 30 s according to the manufacturer’s instructions (Takara, Beijing, China).

### 2.8. Detection of Anti-ASFV Antibodies

The anti-ASFV antibodies in the sera of infected or challenged animals were detected via an indirect enzyme-linked immunosorbent assay (ELISA) targeting the ASFV p54 protein (developed in our laboratory). Briefly, the high-binding 96-well ELISA plates (Corning) were coated with 0.1 μg of purified p54 protein in each well at 4 °C overnight. Plates were washed three times with PBS/T (0.05% (*v*/*v*) Tween-20 in PBS, pH 7.5) and then blocked with 150 μL of 5% skimmed milk at 37 °C for 2 h. After washing three times, 100 μL of diluted samples and controls were added for 1 h of incubation at room temperature (RT). After washing three times, 100 μL of HRP-labeled sheep anti-pig IgG was added for another 1 h of incubation at room temperature. Plates were finally washed and 100 μL of TMB substrate was added for an 8 min colorization at 37 °C. The reaction was stopped using 2 M sulfuric acid at 50 μL/well. The optical density (OD) value was measured at 450 nm. The ratio of OD450 nm of each sample to OD450 nm of positive control was calculated as the S/P value, and S/P > 0.25 is recognized as positive for ASFV antibodies.

### 2.9. Cytokine Assay

Cytokines in sera of infected or challenged animals were detected using the Porcine Cytokine/Chemokine Magnetic Bead Panel Kit (Cat Pcytmag-23K-13px, Millipore, Billerica, MA, USA), and detection was performed strictly according to the instructions supplied by the manufacturer. The types of factors detected and the corresponding lower limits of detection were as follows: IFN-γ: 0.122 ng/mL; IL-1α: 0.005 ng/mL; IL-8: 0.012 ng/mL; IL-4: 0.061 ng/mL; IL-1ra, IL-2, IL-6, IL-10, IL-12, IL-18, TNF-α, and GM-CSF: 0.024 ng/mL. The results were read and analyzed by Luminex®200 and xPONENT® 4.2/4.3 software.

## 3. Results

### 3.1. Construction of the Gene-Deleted ASFV SY18△L7-11

**Figure 1:**
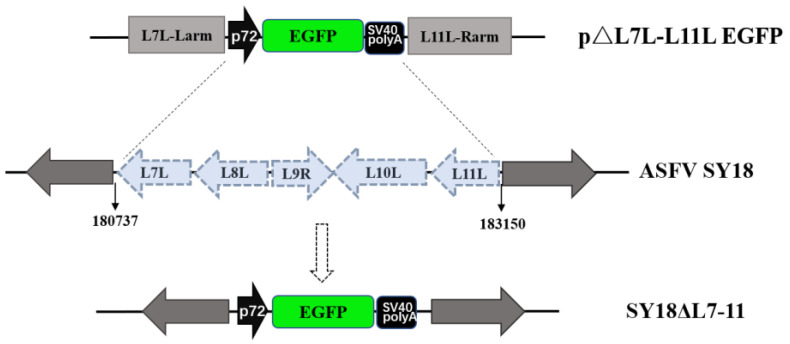
Schematic of recombinant virus construction. The L7L-L11L fragment of African swine fever virus (ASFV) SY18, including the open reading frames (ORFs) of L7L–L11L and the intergenic regions, was replaced by an expression cassette as shown.

The gene deletion mutant was obtained by homologous recombination through co-transfection/infection of BMDM cells with plasmid p△L7L-L11L-EGFP and ASFV SY18. The L7L-L11L fragment of ASFV SY18 was replaced by an expression cassette containing a p72 promoter, an EGFP reporter, and a polyA terminator (Figure 1). After rounds of purification, DNA of the recombinant virus was extracted and PCR identification was performed to confirm no parental strain contamination.

To verify the accuracy of recombination, the full-length sequences of the virus genome were determined using next-generation sequencing (NGS). The results show that the L7L-L11L fragment was successfully replaced and no additional significant mutation and variation were detected when comparing the full-length genome of SY18△L7-11 and parental ASFV SY18 (data not shown).

### 3.2. Replication of SY18△L7-11 In Vitro

**Figure 2:**
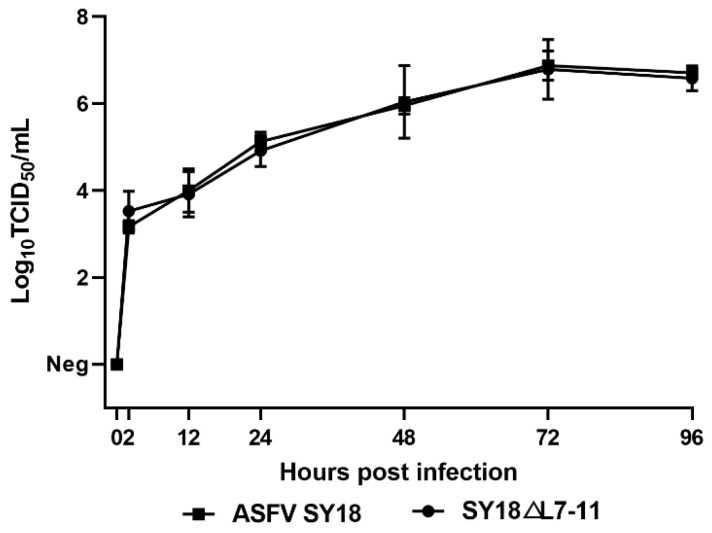
In vitro growth characteristics of SY18△L7-11 and parental ASFV SY18. Primary swine macrophages were infected (multiplicity of infection (MOI) = 0.1) with either SY18△L7-11 or parental ASFV SY18. Samples were taken from three independent experiments at the indicated times postinfection and titrated. Data represent means and standard deviations. Sensitivity of the methodology for this detection is ≥1.625 log_10_ TCID_50_/mL. Neg: negative.

The growth characteristics of SY18△L7-11 were evaluated on BMDMs compared with the parental ASFV SY18. The preformed monolayer BMDMs were infected with either SY18△L7-11 or ASFV SY18 at an MOI of 0.1, and samples were collected at 2, 12, 24, 48, 72, and 96 hpi. The growth curve indicates that the gene-deleted SY18△L7-11 displayed a similar growth kinetic compared to ASFV SY18, as both strains proliferated rapidly once infecting the cells, and reached the maximum titer at 72 hpi (Figure 2). It is shown that deletion of the L7L-L11L fragment of ASFV SY18 did not change the replication ability in vitro.

### 3.3. Virulence of SY18△L7-11 to Swine

**Figure 3:**
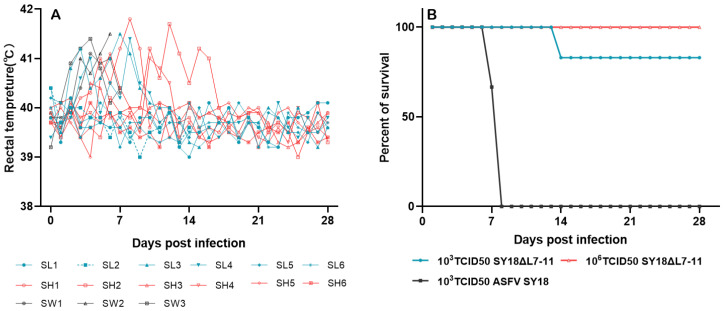
Fever response and survival of swine after inoculation with 10^3^ (SL1-SL6) or 10^6^ (SH1-SH6) TCID_50_ SY18△L7-11 and 10^3^ TCID_50_ ASFV SY18(SW1-SW3). Each curve in figure A represents data from an individual animal. fever response (**A**)and survival (**B**).

To assess the effect on virulence of L7L-L11L deletion, pigs were inoculated intramuscularly (i.m.) with 10^3^ TCID_50_ SY18△L7-11 (Group 1), 10^6^ TCID_50_ SY18△L7-11 (Group 2), or 10^3^ TCID_50_ ASFV SY18 as control (Group 3). As expected, animals in Group 3 exhibited increased body temperature within 3-4 days after the injection, accompanied by diarrhea, anorexia, depression, and skin erythema, and they died or were euthanized on 7–8 dpi. Unlike Group 3, among animals receiving SY18△L7-11, only one pig in Group 1 (SL2, 10^3^ TCID_50_ SY18△L7-11) died on 14 dpi. However, pig SL2 did not show any ASF clinical signs. The other five pigs in Group 1 presented no ASF clinical symptoms, except for transient hyperthermia of two pigs lasting for 2–7 days, and they survived until 28 dpi. As for Group 2 inoculated with 10^6^ TCID_50_ SY18△L7-11, five pigs except SH6 showed transient increase in temperature without other abnormal behaviors of ASF, while one pig named SH2 showed erythema, loss of appetite, and weight loss additionally. Animals in Group 2 all survived for 28 days during the total observation period (Figure 3, Table S2). These results indicate that the recombinant ASFV SY18△L7-11 was less virulent to pigs compared with the parental ASFV SY18.

ASFV genomic DNA in the blood of experimental pigs was quantified at 0, 3, 7, 10, 14, 21, and 28 dpi. As is shown in Table 1, the virus genome in the blood of animals inoculated with 10^3^ TCID_50_ of the virulent parental ASFV SY18 was detected on 3 dpi and continued to rise until the time of death. However, not all animals inoculated with SY18△L7-11 showed viremia. SL2 in Group 1 (died at 14 dpi) presented a growing level of the viremia similar to animals inoculated with the virulent strain but with delayed occurrence, as it was first detected at 7 dpi. SL3 in Group 1 had irregular viremia till the time of challenge, with the other three pigs in the low-dose group showing negative for viremia. As for the high-dose group, only three pigs (SH1, SH2, and SH4) showed irregular viremia, as SH1 presented a long period of low DNA copies till 28 dpi, while copies in the blood of SH2 and SH4 went down from 7 dpi after reaching the highest virus titer (Table 1, Figure S1). 

### 3.4. Protective Effect of SY18△L7-11 against Challenge of Parental ASFV SY18

**Figure 4:**
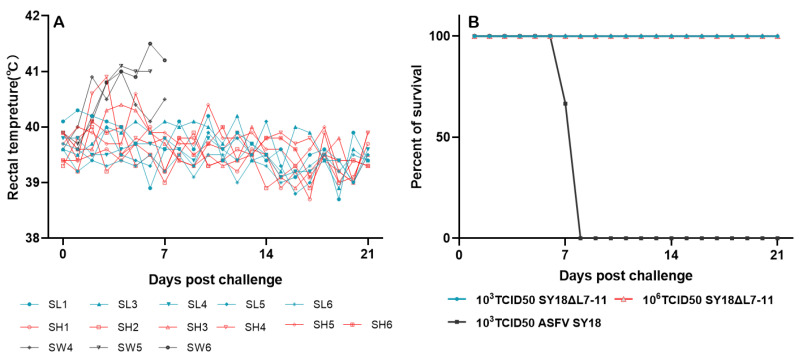
Fever response (**A**) and survival (**B**) of swine after challenge with ASFV SY18. Pigs SL1-SL6 and pigs SH1-SH6 were inoculated with 10^3^ TCID_50_ or 10^6^ TCID_50_ SY18△L7-11 before challenge. Each curve in figure A represents data from an individual animal.

To assess the effect of inoculation with SY18△L7-11 on induction of protection against challenge with parental ASFV SY18, the 11 animals that survived in Experiment 1, including 5 pigs inoculated with 10^3^ TCID_50_ SY18△L7-11 and 6 pigs inoculated with 10^6^ TCID_50_ SY18△L7-11, were challenged with 10^3^ TCID_50_ ASFV SY18 intramuscularly (i.m.) at 28 dpi. Three noninoculated pigs named SW4–SW6 were similarly challenged at the same time as controls (Group 4). The results show that the controls developed typical clinical symptoms of ASF including hyperthermia, depression, anorexia, and diarrhea after challenge and were dead or euthanized at 7–8 dpc. As for the 11 pigs inoculated with SY18△L7-11 before, none of them presented ASFV clinical symptoms until the end of the observation period (21 dpc), except that 2 of them showed transient temperature increases (Figure 4, Table S3).

Viremia was quantified at different time points after challenge. It was found that only 2 of 11 pigs, named SL3 in Group 1 and SH1 in Group 2, were ASFV-p72 positive in the blood, while the rest of the pigs were negative (Table 1). Interestingly, further PCR testing indicated that no L7L-L11L genes were included, showing that the there was no parental ASFV SY18 included (data not shown).

### 3.5. The Immune Response to SY18△L7-11

To assess the immune response of swine to SY18△L7-11, sera of animals experimented were collected every 7 days during the infection and challenging period for antibody detection, and an additional time point of 3 dpi/dpc was added for cytokine detection. 

**Figure 5:**
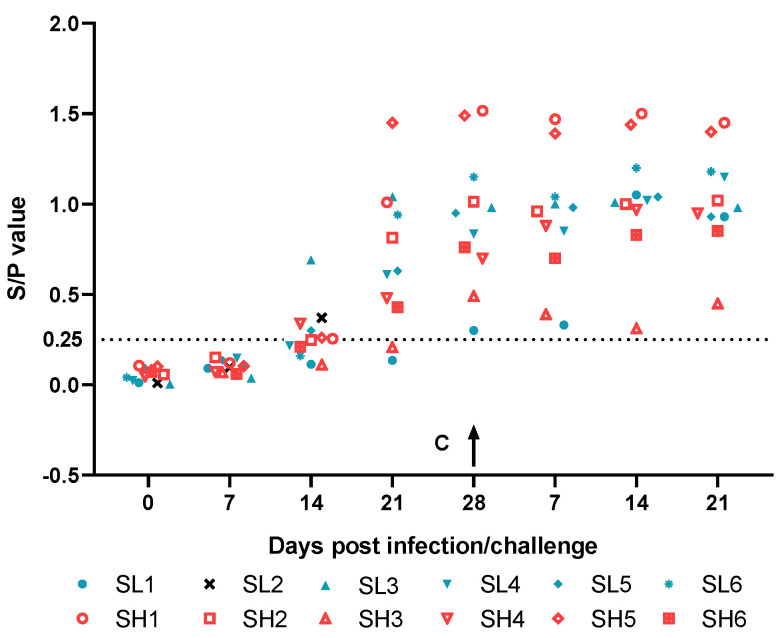
Assessment of ASFV-specific antibodies in pigs inoculated i.m. with 10^3^ (blue, SL1–SL6) or 10^6^ (red, SH1–SH6) TCID_50_ SY18△L7-11 and challenged with 10^3^ TCID_50_ ASFV SY18 at 28 dpi (arrow). The pig SL2 in 10^3^ group which died at 14dpi was shown in black instead of blue. The threshold 0.25 was represented by the dotted line.

Antibody levels are represented as S/P values, and the results show that at 14 dpi, about half of the animals produced anti-p54 antibodies regardless of the immunizing dose. Antibody levels ascended and at 28 dpi, the time of challenge, all pigs exhibited ASFV-specific antibodies according to the threshold of 0.25 (Figure 5). The antibody levels after ASFV SY18 challenge were also detected. The p54 antibody of SL1 in the low-dose group increased from 7 to 14 dpc, reaching the highest S/P value on 14 dpc and then stabilizing. SH4 in the high-dose group exhibited a slight increase in p54 antibodies after challenge. Antibody levels of the rest of the animals were maintained as the day of challenge, also day 28 after inoculation of SY18ΔL7-11. These results indicate that SY18ΔL7-11 can induce animals to produce ASFV-specific antibodies, and it seems there is no correlation between antibody level and vaccination dose.

**Figure 6:**
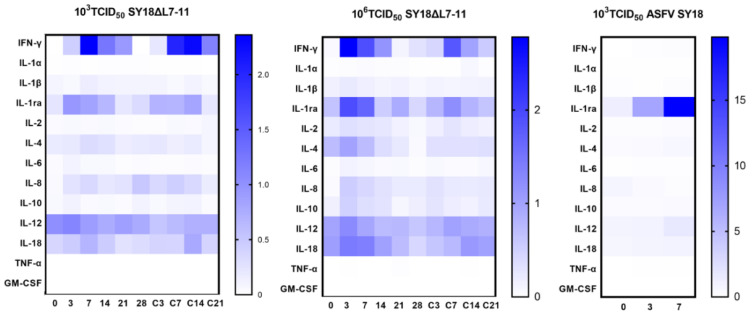
Assessment of cytokines in pigs inoculated i.m. with 10^3^ or 10^6^ TCID_50_ SY18△L7-11 and 10^3^ TCID_50_ ASFV SY18. Animals inoculated with SY18△L7-11 were challenged with 10^3^ TCID_50_ ASFV SY18 at 28 dpi. The heatmap was drawn using the median of each group.

To compare the cytokine kinetics between pigs inoculated with the gene-deletion mutant SY18ΔL7-11 and the parental ASFV SY18, the levels of IFN-γ, IL-1α, IL-1, IL-1ra, IL-2, IL-4, IL-6, IL-8, IL-10, IL-12, IL-18, TNF-α, and GM-CSF in sera of each group after inoculation and challenge were detected using Luminex. The pig SL2 in Group 1 was excluded from data analysis because of its death at 14 dpi; SW2 and SW5 were also excluded for failing to obtain samples at 7 dpi. Thus, SW1, SW3, SW4, and SW6 were included simultaneously as the virulent group. The results show that among pigs inoculated with 10^3^ TCID_50_ SY18△L7-11, 4/5 of the animals presented IFN-γ induction after inoculation. The IFN-γ level peaked at 3–7 dpi (range: 2.36–9 ng/mL), followed by a reduction until challenge. The same trend was observed after challenge. Similarly, six animals inoculated with 10^6^ TCID_50_ SY18△L7-11 exhibited the same trend in IFN-γ, as the level peaked at 3–7 dpi (range: 1.13–8.48 ng/mL) and declined until next rising after challenge. As for pigs inoculated with virulent virus 10^3^ TCID_50_ SY18, sera IFN-γ levels were almost undetectable throughout the infection (Figure 6,  Figure S2A). Levels of each cytokine for individuals are shown in Supplement Figure S2. The level of IL-1ra of animals inoculated with parental ASFV SY18 showed an upward trend, reaching its highest level on the day before death (16.4–22.9 ng/mL). However, the IL-1ra level of animals inoculated with SY18△L7-11 only showed two slight peaks after infection and challenge. The IL-1α, TNF-α, and GM-CSF of the three groups were almost undetectable. Induction of IL-1, IL-2, IL-4, IL-6, IL-8, IL-10, IL-12, and IL-18 was relatively low, and there was no significant difference among the three groups for these cytokines. These results suggest that the mutant strain can induce IFN-γ production, while the parental strain cannot, and the parental strain can induce a higher IL-1ra level before death. It should be noted that pig SL2, which died at 14 dpi in Group 1, induced an extremely high level of IL-1ra (36.59 ng/mL) before death, with almost undetectable IFN-γ, similar to animals inoculated with ASFV SY18.

## 4. Discussion

The ASF epidemic is rapid and widespread in Asia due to a variety of factors, including the active transportation of swine, contaminated meat, disorderly administration of transport vehicles, and lack of experience as this was the first time it hit Asia. The epidemic has become slow and sporadic, probably due to the sharp reduction of pig numbers and the improvement in handling the disease. However, biosafety measures are not effective enough to control the disease in a newly occurring region and with small-scale stockholders. Vaccination is a rational preventive measure. Several vaccine strategies have been put forward [9,12,13], but none have been approved for use. A genetically engineered live ASFV vaccine, with attenuated virulence, is the most feasible approach to serve as an effective ASF vaccine currently [14]. 

The ASFV SY18 strain was isolated from pigs during the first outbreak of African swine fever in China, belonging to genotype II according to the identification on its p72 gene. Our previous work has shown that deletion of CD2v and MGF360/505 in ASFV SY18 could reduce its virulence and provide protection against parental challenge with ASFV SY18 [20]. Deletion of CD2v and UK of the same strain was also reported to be less virulent and capable of providing protection [21]. Deletion of 9GL and UK of HLJ/18, the sequence of which is almost totally same as SY18, reduced the virulence but failed to provide protection [22]. In this study, five consecutive genes located in the variable region at the right end of the genome of ASFV were deleted. None of these genes seem to be involved in the formation of virus particles according to previous reports [23,24]. The potential roles of these genes are not clear, with the exception that the deletion of the L11L gene of Malawi (genotype IX) did not affect the replication or the virulence of the virus [17]. Our results show that the virus could be rescued in primary macrophages after deletion of the five genes without affecting the replication ability, which further indicates that the proteins these genes encode are not key or directly involved in replication and virus assembly.

The degree of viremia of a vaccine virus strain has been reported to be in relation to its protection against challenge in ASFV [9,25]. Although deletion of the L7L-L11L fragment did not affect replication of the virus in vitro, not all of the 12 pigs produced viremia after inoculation with SY18△L7-11, except that 1 out of 12 pigs presented continuously elevated viremia and finally died, 3 out of the remaining 11 pigs showed low levels of viremia, and the vaccine virus was finally cleared before challenge (28 dpi). Two pigs showed long-lasting viremia until the observation period postchallenge, while 6/11 pigs could not be detected with viremia. It is worth noting that ASFV genome was not detected in anal and nasal swabs of each inoculated animal, and neither viremia nor antibodies was detected in sentinels (data not shown). All the above indicate that the frequency and duration of viremia are significantly reduced compared with its parental virus, and the injected virus has only limited replication in vivo. The mechanism of the limited virus replication and where the virus replicates need further elucidation. 

An ideal vaccine candidate for ASFV should have a balance between virulence and immune protection. Some gene-deleted strains may have reduced virulence but may fail to produce protection [26,27]. After deletion of L7L-L11L genes, the SY18△L7-11 could still induce fever in some individuals, even causing death in one pig (SL2) with low-dose inoculation at 14 dpi, and causing anorexia and depression during fever in another pig with high-dose inoculation, with slow growth and weight loss (SH2). This indicates that the virulence of SY18△L7-11 is not reduced enough to be safe for all pigs. However, all 11 animals that survived produced protection against the challenge of virulent ASFV SY18, indicating that this gene-deleted strain may be further improved by codeleting other genes as a vaccine candidate. 

As mentioned at the beginning, we have developed several gene-deleted mutants of ASFV SY18. Animal inoculation trials showed that SY18△CD2v, with the CD2v gene deleted, was still lethal to pigs, while SY18△MGF360/505 (i.e., deletion of MGF360/505) did not produce any clinical symptoms except for mild febrile [20]. SY18△L7-11 constructed in this study could cause a transient increase in body temperature and other side effects after inoculation. Comparing the virulence, SY18△L7-11 is moderate between SY18△CD2v and SY18△MGF. As additional deletion of MGF360/505 on SY18△CD2v could further attenuate its virulence and maintain immunity to a challenge, we suppose that additional deletion of other genes in SY18△L7-11 may generate a safer and more effective vaccine candidate. Furthermore, as SY18△L7-11 contains five consecutive ORFs, one or more genes in L7L-L11L may play a key role in attenuating the virulence. Hence, it is necessary to further address the function and attenuation mechanisms for each gene.

The mechanism of immunoprotection stimulated by attenuated ASFV is a complex cascade. Circulating antibodies to ASFV may play a key role. Evidence suggests that passive transfer of antibodies from immunized pigs to immature pigs may provide protection against the virus [28]. However, the presence of circulating virus-neutralizing antibodies in animals inoculated or infected with attenuated ASFV strains has been a controversial issue [13]. In order to check the antibodies in each animal, we conducted a specific antibody assay against the p54 protein. The results show that all the animals that survived the inoculation produced antibodies before the challenge. Notably, antibody production was independent of viral load in the blood, as antibodies to p54 were even present in animals without any viremia. 

Type I IFN is usually supposed to play an important role in inhibition of ASFV replication [8,13,29]. However, the role of IFN-γ in ASFV infection is controversial. For example, vaccination with the attenuated OURT88/3 strain was reported to induce high numbers of IFN-γ-producing lymphocytes in vivo [8]. For Benin△MGF, the induction of IFN-γ might influence the delay appearance of clinical signs and the onset of death in non-protected pigs after a challenge [30]. However, the IFN-γ T cell response to the attenuated Benin strains was different depending on the genes deleted, indicating that other T cell responses may exist in addition to IFN-γ production [29,30]. Moreover, the IFNs seem to have relations with the limited replication of attenuated strains in vivo [31,32]. SY18△L7-11 could induce IFN-γ, which is the opposite of its parental strain ASFV SY18. That is to say, the inhibition of ASFV SY18 on IFN-γ production was diminished due to the deletion of L7L-L11L, indicating that one or more of the five genes may exert an effect on interferon inhibition for the virus.

IL-1ra competitively binds to the interleukin-1 receptor and subsequently blocks the intracellular IL-1 signaling cascade. Therefore, it is an early anti-inflammatory cytokine that controls inflammatory responses during an early stage of immune activation. In four pigs inoculated with ASFV SY18 and one pig inoculated with SY18△L7-11 followed by dying at 14 dpi, IL-1ra was significantly elevated at 3–7 dpi (Figure S2). However, in other pigs inoculated with SY18△L7-11, no significant change was observed regarding the level of IL-1ra. The elevation of IL-1ra has been described in several infectious diseases, but the role and potential benefits or detriments of IL-1ra are unclear. A delicate balance exists between the beneficial effects of pathogen clearance and the detrimental effects of an overactive immune response. Early IL-1ra production was reported to negatively modulate proinflammatory cytokine and type I IFN production during the early phase of PRRSV infection [33]. In Ebola virus infection, a high level of IL-1ra was found to contribute to the protection of the host [34]. In this study, we supposed that IL-1ra may play a role in anti-inflammatory response when a lethal disease develops, while inoculation with SY18△L7-11 stimulates less inflammation, thus hardly inducing an anti-inflammatory effect. However, levels of other anti-inflammatory cytokines, such as IL-4 and IL-10, and proinflammatory cytokines in the animals were barely detected; thus, we failed to find more relations between the pro- and anti-inflammatory responses. This may be due to the limited sensitivity of the testing kit and the time intervals between each sample collection. More evidence is needed in the future to support this hypothesis. 

## 5. Conclusions

In conclusion, we obtained an attenuated strain (SY18△L7-11) by means of homologous recombination. It produced irregular viremia in swine and led to a mild clinical presentation with one dose of inoculation, as the virulence was obviously dramatically reduced. Inoculation with SY18△L7-11 eventually stimulated protective immunity against the challenge by its homologous parental virulent strain ASFV SY18. Thus, the genes L7L-L11L are newly found virulence determinants of ASFV. In view of its side effects, such as mild fever, it is necessary to further codelete one or more genes to improve its safety if a vaccine candidate is desired.

## Figures and Tables

**Table 1:** Virus genome copies in blood samples of swine after inoculation or challenge.

Groups	No.	ASFV Genome Copies/mL (log_10_)											
		Days Postinoculation						Days Postchallenge					
		0	3	7	10	14	21		28	3	7	14	21
10^3^ TCID_50_ SY18△L7-11	SL1	- ^a^	-	-	-	-	-		-	-	-	-	-
	SL2	-	-	-	6.97	7.87	/ ^b^						
	SL3	-	6.29	6.70	5.79	-	5.12		5.39	5.44	5.19	4.48	3.62
	SL4	-	-	-	-	-	-		-	-	-	-	-
	SL5	-	-	-	-	-	-		-	-	-	-	-
	SL6	-	-	-	-	-	-		-	-	-	-	-
10^6^ TCID_50_ SY18△L7-11	SH1	-	5.35	-	5.12	3.88	5.42		4.88	3.76	3.85	-	-
	SH2	-	6.07	7.42	7.08	5.33	4.98		-	-	-	-	-
	SH3	-	-	-	-	-	-		-	-	-	-	-
	SH4	-	5.85	7.25	5.06	-	5.03		-	-	-	-	-
	SH5	-	-	-	-	-	-		-	-	-	-	-
	SH6	-	-	-	-	-	-		-	-	-	-	-
10^3^ TCID_50_ ASFV SY18	SW1	-	5.62	9.37	/								
	SW2	-	7.30	/									
	SW3	-	5.47	8.34	/								
	SW4								-	6.37	8.71	/	
	SW5								-	7.20	/		
	SW6								-	7.57	9.10	/	

^a^ -: negative, ^b^ /: dead.

## Data Availability

The data presented in this study are available in the present article and in supplementary material.

## Supplementary Materials

The following are available online at https://www.mdpi.com/1999-4915/13/2/255/s1, Figure S1: Virus genome copies in blood samples of swine inoculated i.m. with 10^3^ (SL1-SL6) or 10^6^ (SH1-SH6) TCID_50_ SY18△L7-11 and 10^3^ TCID_50_ ASFV SY18 (SW1-SW6), Figure S2: Cytokines of individual pigs inoculated i.m. with 10^3^ (SL1–SL6) or 10^6^ (SH1–SH6) TCID_50_ SY18△L7-11, and 10^3^ TCID_50_ ASFV SY18 (SW1–SW6). Table S1: Primers for (quantitative) PCR assay recommended by the national standards, Table S2: Survival and fever response of swine after inoculation with SY18△L7-11 or parental ASFV SY18. Table S3: Survival and fever response of swine after challenge with ASFV SY18.
