# Supplementary material for: Deletion of the L7L-L11L Genes Attenuates ASFV and Induces Protection against Homologous Challenge
Source: Viruses. 2021 Feb 8;13(2):255. doi: 10.3390/v13020255 (PMC7915138; doi:10.3390/v13020255)
Supplement: Supplementary file 1 [file viruses-13-00255-s001.zip › supplementary materials V3.0/Supplement materials V3.0.docx]

**Table S1.** Primers for (quantitative) PCR assay recommended by the national standards

| **Viruses** | **Primers** | **Sequences (5’-3’)** | **Product size(bp)** | **No. of reference standard** |
| --- | --- | --- | --- | --- |
| CSFV | CSFV-qF | TACAGGACAGTCGTCAGTAGTTCGA-3’ | /^a^ | GB/T 27540-2011 |
|  | CSFV-qR | CCGCTAGGGTTAAGGTGTGTCT |  |  |
|  | CSFV-qP | FAM-CCCACCTCGAGATGCTATGTGGACGA-TAMRA |  |  |
| PRRSV | PRRSV-P_1_ | GGTTCGGAAGAAACTGTCGG | HP^b^: 400  LP^c^: 264 | GB/T 27517-2011 |
|  | PRRSV-P_2_ | AGCAGGTGGAAGAAGCGAATC |  |  |
|  | PRRSV-P_3_ | GAGCTGAGTATTTTGGGCGTG |  |  |
| PPV | PPV-F | TGGTCTCCTTCTGTGGTAGG | 445 | SN/T 1874-2007 |
|  | PPV-R | CAGAATCAGCAACCTCAC |  |  |
| PRV | PRV-gD-P_1_ | CAGGAGGACGAGCTGGGGCT | 217 | GB/T 18641-2018 |
|  | PRV-gD-P_2_ | GTCCACGCCCCGCTTGAAGCT |  |  |
| PCV | PCV-P_1_ | CCGCGGGCTGGCTGAACTT | PCV-1: 652  PCV-2: 1154 | GB/T 21674-2008 |
|  | PCV-P_2_ | CTCGGCTATGCGCTCCAAAATG |  |  |
|  | PCV-P_3_ | ACCCCCGCCACCGCTACC |  |  |

^a^ /: real time quantitative PCR

^b^ HP: high pathogenic

^c^ LP: low pathogenic

**Table S2.** Survival and fever response of swine after inoculation with SY18△L7-11 or parental ASFV SY18.

| **Groups** | **Viruses** | **No. of**  **survivors/total** | **Clinical signs** | | |
| --- | --- | --- | --- | --- | --- |
|  |  |  | **No. of pigs with**  **fever/total** | **Duration (day)** | **Highest body**  **temperature (°C)** |
| 1 | 10^3^ TCID_50_ SY18△L7-11 | 5/6 | 2/6 | 7 | 41.5 |
| 2 | 10^6^ TCID_50_ SY18△L7-11 | 6/6 | 5/6 | 2-7 | 41.8 |
| 3 | 10^3^ TCID_50_ ASFV SY18 | 0/3 | 3/3 | 3-5 | 41.5 |

**Table S3.** Survival and fever response of swine after challenge with ASFV SY18.

| **Virus**  **immunized** | **Virus**  **Challenged** | **No. of**  **survivors/total** | **Clinical signs** | | |
| --- | --- | --- | --- | --- | --- |
|  |  |  | **No. of pigs with fever/total** | **Duration (day)** | **Highest body**  **temperature (°C)** |
| 10^3^ TCID_50_ SY18△L7-11 | 10^3^ TCID_50_ ASFV SY18 | 5/5 | 0/5 | 0 | 40.3 |
| 10^6^ TCID_50_ SY18△L7-11 |  | 6/6 | 2/6 | 1-2 | 40.9 |
| Mock |  | 0/3 | 3/3 | 4-5 | 41.5 |


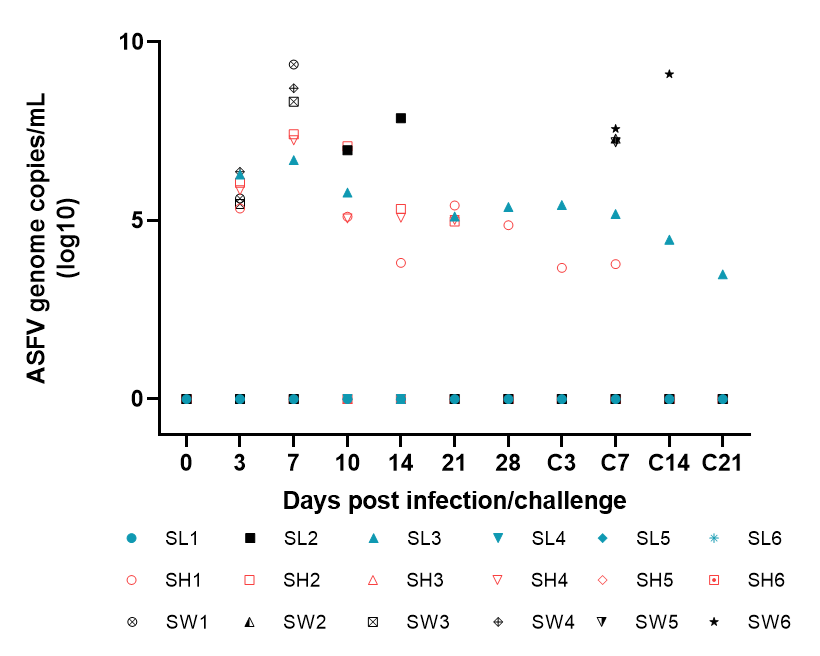


**Figure S1.** Virus genome copies (per mL) in blood samples of swine inoculated i.m. with 10^3^ (SL1-SL6) or 10^6^ (SH1-SH6) TCID_50_ SY18△L7-11 and 10^3^ TCID_50_ ASFV SY18(SW1-SW6). Animals inoculated with SY18△L7-11 were challenged(C) with 10^3^ TCID_50_ ASFV SY18 at 28 dpi.


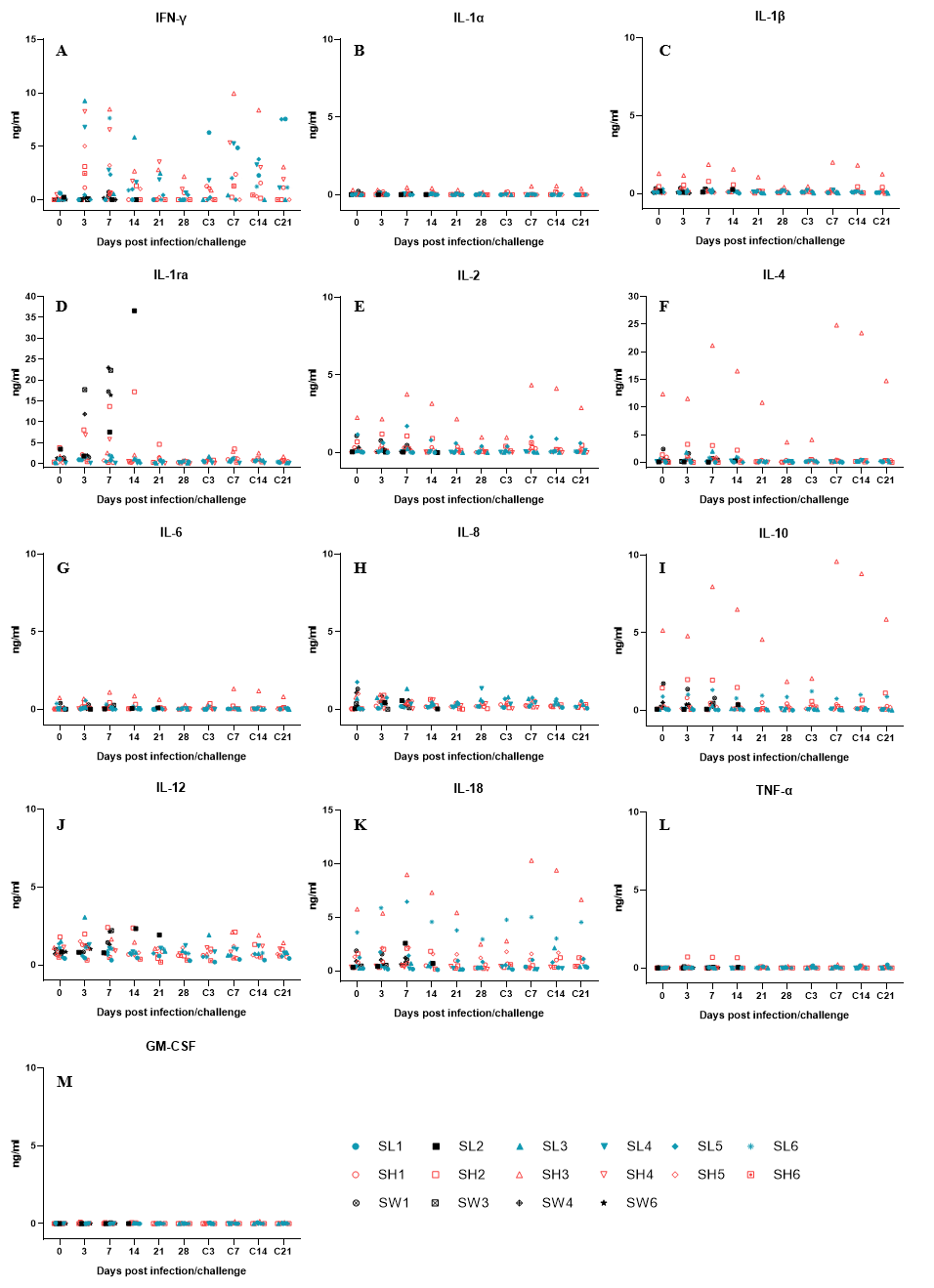


**Figure S2.** Cytokines of individual pigs inoculated i.m. with 10^3^ (SL1-SL6) or 10^6^ (SH1-SH6) TCID_50_ SY18△L7-11 and 10^3^ TCID_50_ ASFV SY18 (SW1-SW6). Animals inoculated with SY18△L7-11 were challenged(C) with 10^3^ TCID_50_ ASFV SY18 at 28 dpi.
