# Supplementary figures and images for: Deletion of the L7L-L11L Genes Attenuates ASFV and Induces Protection against Homologous Challenge
Source: Viruses. 2021 Feb 8;13(2):255. doi: 10.3390/v13020255 (PMC7915138; doi:10.3390/v13020255)

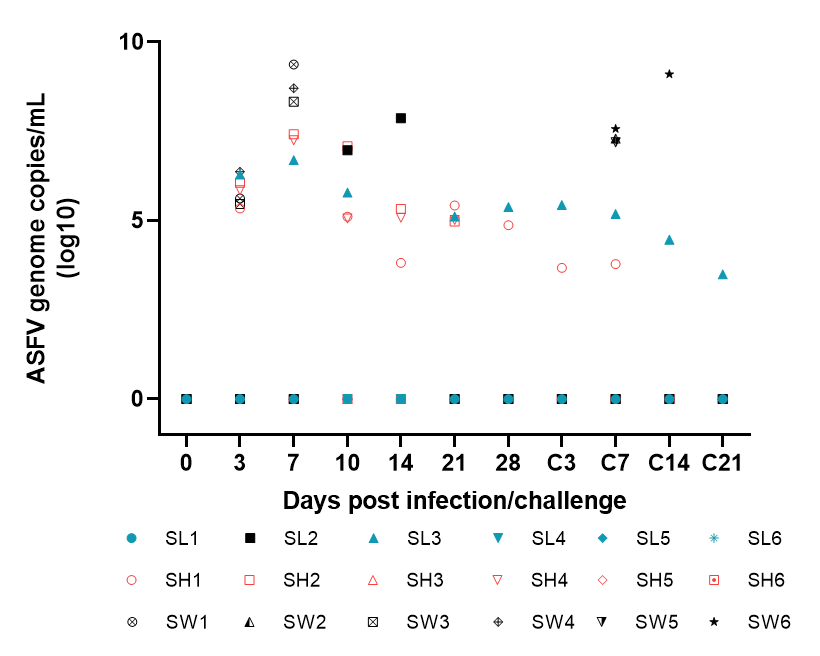

Supplement: Supplementary file 1 [file viruses-13-00255-s001.zip › supplementary materials V3.0/Figure S1.tif]

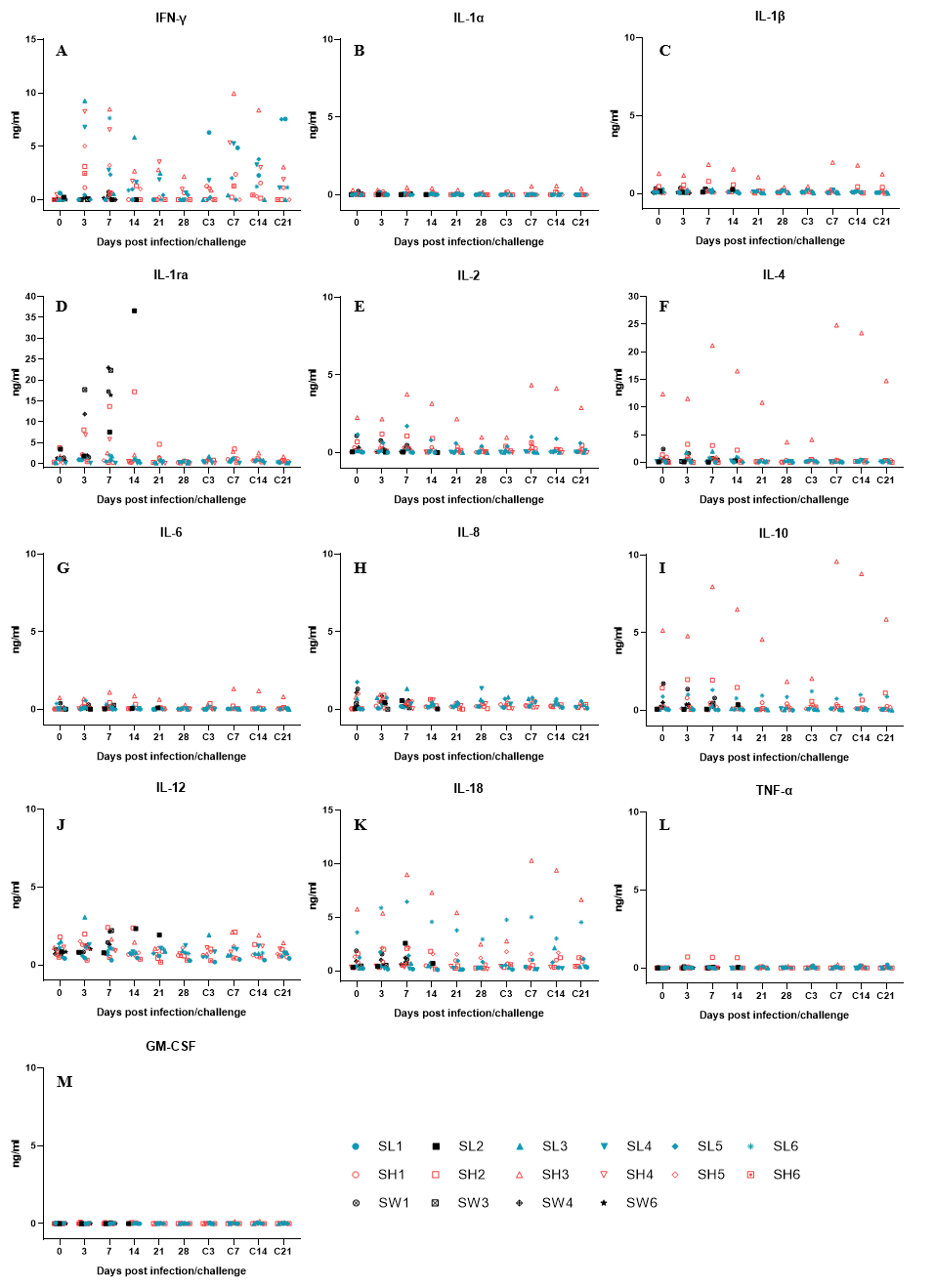

Supplement: Supplementary file 1 [file viruses-13-00255-s001.zip › supplementary materials V3.0/Figure S2.tif]
